# Blood pressure characteristics in patients with acute basilar artery occlusion undergoing endovascular thrombectomy

**DOI:** 10.1038/s41598-019-49769-8

**Published:** 2019-09-13

**Authors:** Slaven Pikija, Katharina Millesi, Monika Killer-Oberpfalzer, J. Sebastian Mutzenbach, Laszlo K. Sztriha, Michael U. Füssel, Johann Sellner

**Affiliations:** 1 Department of Neurology, Christian Doppler Medical Center, Paracelsus Medical University, Salzburg, Austria; 2 Research Institute for Neurointervention, Christian Doppler Medical Center, Paracelsus Medical University, Salzburg, Austria; 3 Department of Neurology, King´s College Hospital, Denmark Hill, London, United Kingdom; 4 Institute of Neuroanesthesiology, Christian Doppler Medical Center, Paracelsus Medical University, Salzburg, Austria; 5 Department of Neurology, Klinikum rechts der Isar, Technische Universität München, München, Germany

**Keywords:** Stroke, Stroke

## Abstract

Acute basilar artery occlusion (BAO) is a rare but potentially life-threatening neurological condition. While endovascular therapy (EVT) has been shown to improve outcome, there is limited knowledge about prognostic factors beyond early recanalization. We studied whether blood pressure (BP) exceeds or falls below suggested thresholds during intervention and whether these changes are associated with complications and outcome. BP measurements mostly with one-minute intervals were available in 39 patients. An individual systolic blood pressure (SBP) reference value was defined as the median of the first five intra-procedural measurements. Half of the patients (51.3%) received drugs for BP augmentation and two a BP lowering drug (5.1%). Thrombolysis in cerebral infarction grade 2b and 3 (TICI) was achieved in 29 (74.4%) and 23 patients (58.9%) had good outcome at three months. We observed a continuous intra-procedural increase of median SBP (+11%) and mean arterial pressure (MAP, +10%, both p < 0.001), and a unique temporal pattern of intermittent peaks and troughs. Successful recanalization was more common in patients whose intra-procedural duration with SBP under 140 mmHg was shorter (p = 0.009). Patients with isolated tip of basilar artery (TBA) occlusion had significantly more BP excursion of 20% below the reference SBP and required more frequent use of sympathomimetic drugs compared to vertebrobasilar occlusion (p = 0.008 and p = 0.041, respectively). Brain hemorrhage was more prevalent in patients who experienced SBP excursions at least 20% above the individual reference value (p = 0.038) and a longer duration of time spent with SBP above 180 mmHg (p = 0.029). Patients with higher pre-procedural mean SBP had a greater chance of a good outcome (p = 0.03). This study using high resolution BP monitoring suggests a relationship between intra-procedural BP characteristics and recanalization, hemorrhagic complications and outcome in patients receiving EVT for acute posterior circulation cerebrovascular syndromes. Differences with regard to BP regulation during recanalization therapy for vertebrobasilar and TBA occlusion deserves further attention.

## Introduction

Acute basilar artery occlusion (BAO) is a rare but devastating disorder with mortality rates of up to 95% if recanalization cannot be achieved^1,2^. The recognition of this life-threatening condition is frequently hindered by a stuttering clinical course, unspecific symptoms and potential for rapid decline. With endovascular therapy (EVT), which removes the clot by using a microcatheter device or aspiration technique, about one third of patients can reach independent outcome defined as a modified Rankin Scale (mRS) of 0–2^3,4^. Notably, in contrast to anterior circulation ischemic stroke there is a lack of randomized-controlled trials confirming the benefit of EVT for BAO^5^. It is reasonable, however, to perform EVT in the setting of acute BAO based on the currently available evidence^2^. The continued disease burden despite reperfusion success in BAO is illustrated by a study of 212 consecutive patients between 2011 and 2017, which revealed rates for recanalization and mortality of 91.5% and 16%, respectively^6^. Moreover, there was a significant rate of intracranial hemorrhages (7.6%). Early and successful recanalization have been identified as the most important predictive factors for good outcome in acute BAO^7,8^. Interestingly, Shu *et al*. reported that successful recanalization does not correlate with thrombus length^9^. Moreover, failure of successful recanalization has been recognized as a strong predictor for mortality^10^. This is highlighted by a recent meta-analysis which showed that recanalization was associated with a 1.5-fold reduction in the risk of death or dependency, and a 2-fold reduction in the risk of mortality^11^. Additional variables include the best method of recanalization (intra-arterial thrombolysis, mechanical thrombolysis, or a combination), the time window for the treatment, and patient selection.

There is emerging evidence from large vessel occlusion in the anterior circulation that not only successful recanalization but also intra-procedural factors contribute to outcome. Despite the large body of literature on EVT in stroke, there is only scarce research on the most appropriate anesthetic or hemodynamic management during these procedures as well as temperature control and type of anesthesia. Current guidelines including the recommendations from the Society for Neuroscience in Anesthesiology and Critical Care propose that a systolic BP (SBP) should be maintained between 140 and 180 mmHg in patients who are candidates for thrombolytic or endovascular therapy^12,13^. As these recommendations are mainly based on evidence derived from studies in anterior circulation ischemic stroke, the findings may not generalizable for the posterior circulation^14–21^. Moreover, there is limited knowledge about occurrence of extremes and variability of BP in large vessel occlusion of the posterior circulation^22^. The purpose of the present study was thus to examine whether BP exceeds or drops below suggested thresholds during EVT for acute BAO and whether these BP changes are associated with complications and outcome.

## Subjects and Methods

We performed a retrospective study of all consecutive stroke patients admitted to Christian Doppler Medical Center (Salzburg, Austria) from January 2011 to October 2018. We searched for patients with acute vertebrobasilar occlusion confirmed on CT – or MR-angiography. Exclusion criteria were age under 18 years, hemorrhage on baseline neuroimaging, lack of BP measurement data and poor neuroimaging quality. The brain images on admission and follow-up were evaluated by a vascular neurologist blinded to outcome (KM). Vessel occlusion was grouped to basilar artery with or without vertebral artery occlusion (BAO) and to isolated tip of basilar artery (TBA) occlusion.

EVT was performed with modern stent-retriever or aspiration devices in all patients according to in-house protocols. Prior to EVT the patients underwent anesthesiologic assessment and received a device for BP monitoring. The invasive and non-invasive BP measurements were recorded once per minute or every five minutes, respectively. The recordings were processed in MetaVision® (IMDSoft, Düsseldorf, Germany) together with details of other vital parameters, medication and anesthesiologic procedures. The entire set of BP measurements was analyzed in three groups: pre-procedural (of short duration), intra-procedural (during intervention) and post-procedural (time from sealing of the femoral artery until transfer to the ICU). While the in-house protocol suggested avoidance of excessive hypotension (MAP < 65 mmHg) or excessive hypertension (MAP > 130 mmHg), utilization of medication was at the discretion of the anesthesiologist. From the angio-CT suite all patients were transferred to the neurological ICU for further observation. Follow-up CT or MRI was performed within 24 hours and in most cases at least once thereafter. The last available image (but not older than 30 days after stroke) was assessed for analysis of a potential ischemic lesion. The regions of interest included brainstem, cerebellum, thalamus and the region supplied by the posterior cerebral artery. In addition, the presence of intra-procedural hemorrhage was assessed. Medical charts were studied for patient demographics, cause of stroke, pre-morbid mRS and medication. Good outcome was defined as Barthel index ≥ 50 at discharge or transfer to a rehabilitation facility.

An individual reference SBP was determined as the median of the first five procedural measurements. Of note, the subsequent time period required for obtaining this reference value was 5 min. for invasive and 25 min. for non-invasive BP determination. From this reference SBP, we calculated the number of intra-procedural excursions 20% below and 20% above this threshold, and duration of these excursions. When not stated otherwise, the analysis pertains to intra-procedural BP values.

Following BP indices were calculated: maximum to minimum SBP difference (MMD), standard deviation (SD), coefficient of variation in % (CV; SD/mean (BP)*100). Additionally, to better assess for changes over short time intervals, we used average real variability (ARV) which was calculated using the formula proposed by Mena *et al*.:

$$ARV=\frac{1}{N-1}\mathop{\sum }\limits_{k=1}^{N-1}|B{P}_{k+1}-B{P}_{k}|$$

where N denotes the number of valid BP measurements, and k is the order of measurements^23^.

## Statistical Methods

Since there were no normally distributed continuous variables as tested by Shapiro-Wilk test, we used the non-parametric Kruskal-Wallis test. We used Fisher´s exact test for group comparisons. We dichotomized categorical variables such as age (equal or greater to 65 years vs. younger), TICI outcome (0–2a vs. 2b and 3), clinical presentation at admission (conscious vs. intubated). Mean pre-procedural SBP was calculated and used in multiple comparison model. To compare groups with multiple measurements of pre-procedural SBP in individual patients, we used Kenward-Rogers approximation for degrees-of-freedom in order to obtain p-values. In this instance, we compared mean SBP values. The continuous variables were also transformed when skew were present (square root or natural logarithm). The P value was set at <0.05. The statistical analysis was performed using R statistical Software (R Core Team, Vienna, Austria)^24^.

## Results

### Cohort

We identified 47 patients with angiographically confirmed acute BAOs. Of them, 39 underwent EVT and had BP records saved in MetaVision (iMDSoft, Düsseldorf, Germany). The demographics of the cohort are shown in Table 1. The median NIHSS on admission was 16.0 (interquartile range IQR 6.5–42.0) and 13 patients (33.3%) arrived at the emergency room in sedated and intubated condition. EVT was performed in conscious sedation in five patients and three patients had non-invasive BP measurement (12.8% and 7.7%, respectively). There were no unusual observations with regard to the first five BP measurements in the three patients in whom BP was determined non-invasively. The drugs used for induction of anesthesia included propofol, fentanyl and rocuronium. Bridging with alteplase was performed in 22 patients (56.4%). Thrombolysis in cerebral infarction grade 2b and 3 (TICI) was achieved in 29 (74.4%). Good outcome (Barthel score >50) was present in 23 patients (59.0%) at 3 months.

**Table 1:** Baseline demographics, data on intervention and outcome in 39 patients treated with EVT for posterior circulation ischemic stroke.

	All (N = 39)
**Age**, **years**	73.5 (59.3–81.5)
**Age ≥65 years**	28 (71.8)
**Women**	14 (35.9)
**Pre-mRS** (**0–2**)	33 (84.6)
**Clinical outcome: good** (**Barthel Index ≥50**)	23 (58.9)
**Medical history**	
Stroke/TIA	7 (17.9)
Arterial hypertension	28 (71.8)
Hyperlipidemia	12 (30.8)
Diabetes	3 (7.7)
ICA Stenosis ≥50%	2 (5.1)
Antiplatelet drugs	11 (28.2)
Anticoagulants	3 (7.7)
Statins	10 (25.6)
**TOAST stroke etiology**	
Cardioembolic	19 (48.7)
Large artery atherosclerotic	6 (15.4)
Other	4 (10.3)
Unknown	10 (25.6)
**Laboratory on admission**	
Glucose mg/dl	130.0 (115.0–152.0)
LDL mg/dl	90.0 (78.0–106.0)
Erythrocytes 10^6^/µL	4.6 (4.3–4.8)
**NIHSS on admission**	16.0 (6.5–42.0)
**Intubated on admission**	13 (33.3)
**Time to admission in minutes**	75.0 (46.0–180.0)
**Vessel occlusion**	
Vertebrobasilar	26 (66.7)
Tip of the basilar artery	13 (33.3)
Bridging with alteplase	22 (56.4)
**Interventional data**	
Duration in minutes	72.0 (49.5–115.5)
General anesthesia	34 (87.2)
Invasive RR measurement	36 (92.3)
BP augmentation	20 (51.3)
BP lowering	2 (5.1)
Both BP drugs given	1 (2.6)
No BP drugs given	17 (43.5)
TICI outcome (2b and 3)	29 (74.4)
**Neuroimaging outcome: location of infarction or presence of hemorrhage**	
Brainstem	18 (46.2)
Cerebellar	24 (61.5)
Posterior territory	18 (46.2)
Thalamus	12 (30.8)
Parenchymal hemorrhage	4 (10.3)
No stroke visible	6 (15.3)

Legends: Absolute number (%) or median and IQR. mRS – modified Rankin scale, TICI – thrombolysis in cerebral infarction sale, BP – blood pressure, IV – intravenous, TOAST – Trial of Org 10172 in Acute Stroke, LDL – low density lipoprotein, ICA – internal carotid artery, pre-mRS – mRS scale before stroke.

The median duration of procedure was 72 minutes (IQR 49.5–115.5), as shown in Table 2. Median CV was 9.6 (IQR 5.5–12.7) and the MMD was 57.4 mmHg (IQR 28.5–78.0). Intra-procedural MMD was higher in patients ≥65 years with a median of 65.0 mmHg (IQR 33.2–88.0) compared to patients aged <65 years with a median of 37.0 mmHg (IQR 20.5–36.5, respectively), p = 0.0419)).

**Table 2:** Characteristics of systolic and mean arterial blood pressure before endovascular thrombectomy (pre-procedural) (EVT), during intervention (intra-procedural) and thereafter (postprocedural) in 39 patients treated due to acute basilar artery occlusion.

	Pre-procedural, N = 496	Intra-procedural, N = 3103	Post-procedural, N = 469
Number of measurements	11.5 (8.25–22.5)	72.0 (38.5–116.5)	11.0 (4.0–18.2)
SBP, mmHg	122.0 (107.0–143.0)	135.0 (118.0–151.0)	128.0 (117.0–139.0)
MAP, mmHg	84.0 (75.8–96.2)	92.0 (81.0–101.0)	91.0 (79.0–99.0)

Legends: Data are median (interquartile range). SBP – systolic blood pressure. MAP – mean arterial pressure. There were 9 and 7 missing measurements for pre- and post-interventional SBP and MAP, respectively.

### Type of basilar artery occlusion and location of ischemic infarcts

Brainstem infarcts were significantly more frequent in vertebrobasilar than in isolated TBA occlusion. Specifically, 15/18 (83.3%) of brainstem infarcts were related to vertebrobasilar occlusion, while 10/19 (52.6%) of non-brainstem infarcts were caused by isolated TBA occlusion (p = 0.038). The procedure duration did not differ between BA vs. tip of BA occlusion and between various infarct locations.

### Interventional epoch

**Figure 1:**
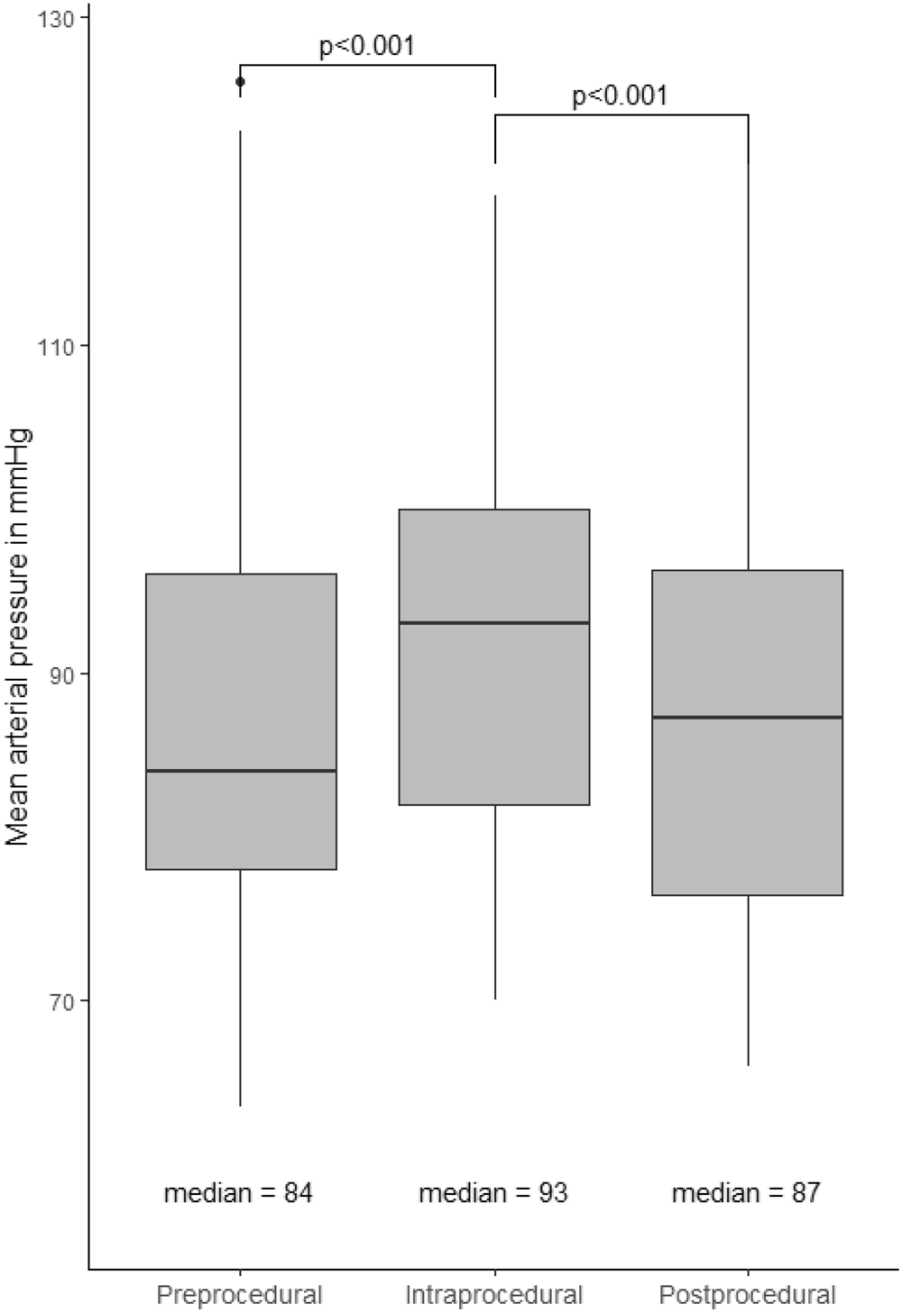
Comparison of median MAP prior to, during and after EVT.

Median SBP and MAP prior to EVT were 122.0 mmHg (IQR 107.0–143.0) and 84.0 mm Hg (75.8–96.2), respectively, as shown in Fig. 1. Median intra-procedural SBP and MAP were 135.0 (IQR 118.0–151.0) and 92.0 (IQR 75.0–93.0) mmHg, respectively. The levels during intervention were significantly higher compared to pre-interventional levels for both SBP and MAP (rise of 11% and 10%, respectively; p < 0.001). BP did not return to reference levels in the immediate post-procedural setting (SBP 128.0 mmHg (IQR 117.0–139.0) and MAP 91.0 mmHg (79.0–99.0), p < 0.001)).

There were 19 patients (49%) who had SBP excursions 20% or more above the reference SBP and 12 patients (31%) who had SBP excursion <20% below the reference SBP in the course of their EVT procedure.

Patients with isolated TBA occlusion spent significantly longer time with SBP 20% below their individual reference value (mean 15.2 vs. 0.73 minutes, p = 0.001), and received more sympathomimetic drugs than patients with BAO (p = 0.041). Significantly more patients with TBA occlusion (8/13, 61.5%) had excursions 20% below the reference SBP compared to patients with BAO (4/22, 15.4%, p = 0.008). A similar proportion of patients with vertebrobasilar (50%) and TBA (46.2%) had excursions 20% above the SBP reference value (p = 0.86).

### Medication

The usage of sympathomimetic drugs showed positive weak association with ARV p = 0.054, and stronger with SD and MMD (p < 0.01 and p < 0.01, respectively), as shown in Table 3. Moreover, patients receiving BP raising medication had a longer duration of SBP below 20% of reference values (mean 10.4 minutes when used vs. 0.42 minutes when not used, p = 0.021). Yet, patients in whom sympathomimetic drugs were administered had a trend for a better outcome (p = 0.054).

**Table 3:** Systolic blood pressure parameters in 39 patients during endovascular intervention for acute posterior circulation ischemic stroke stratified for patients with and without BP augmenting drugs.

	All	BP aug. drug (N = 20)	No BP aug. drug (N = 19)	P
Standard deviation	12.9 (7.4–16.0)	17.1 (12.4–19.5)	8.4 (4.8–10.1)	<0.001
Coefficient of variation	9.6 (5.5–12.7)	12.7 (8.9–15.1)	6.2 (4.4–7.5)	<0.001
Average real variability	4.0 (2.5–4.7)	4.7 (3.1–5.4)	3.3 (2.2–4.0)	0.053
Maximum-minimum difference, mmHg	57.4 (28.5–78.0)	76.1 (58.5–95.2)	37.3 (20.5–50.5)	<0.001
Number of excursions ≥120% SBP	13.6 (0.0–13.0)	13.1 (0.0–14.8)	14.1 (0.0–9.0)	0.608
Time spent ≥120% SBP	14.1 (0.0–15.0)	14.1 (0.0–23.2)	14.2 (0.0–9.0)	0.556
Number of excursions ≤80% SBP	4.8 (0.0–2.0)	9.0 (0.0–10.8)	0.5 (0.0–0.0)	0.024
Time spent under ≤80% SBP	4.8 (0.0–2.0)	9.0 (0.0–10.8)	0.5 (0.0–0.0)	0.011
Number of SBP excursions <140 mmHg	45.9 (14.5–75.0)	49.7 (17.8–79.5)	41.8 (4.0–69.0)	0.399
Time spent under 140 mmHg	48.4 (18–76.0)	51 (24.5–79.8)	45.7 (14.5–73.0)	0.643
Number of SBP excursions <100 mmHg	3.0 (0.0–3.5)	1.5 (0.0–5.0)	0.0 (0.0–1.5)	0.121
Time spent under 100 mmHg	3.0 (0.0–3.5)	4.4 (0.0–5.0)	1.6 (0.0–2.0)	0.089
Number of SBP excursions ≥180 mmHg	3.7 (0.0–5.0)	4.8 (0.0–5.2)	2.6 (0.0–4.5)	0.295
Time spent ≥180 mmHg	2.4 (0.0–2.0)	3.45 (0.0–2.0)	1.31 (0.0–0.5)	0.356

SBP – systolic blood pressure, aug. – augmention, low. – lowering. Data are presented as median (interquartile range).

Administration of BP lowering drugs was positively associated with SBP SD, COV, ARV, MMD in mmHg, number of measurements of SBP over 180 mmHg (p = 0.030, p = 0.048, p = 0.048, p = 0.041 and p = 0.008, respectively). However, the number of patients were small, with only two patients receiving BP lowering medication.

### Correlations of BP measures and other variables

**Figure 2:**
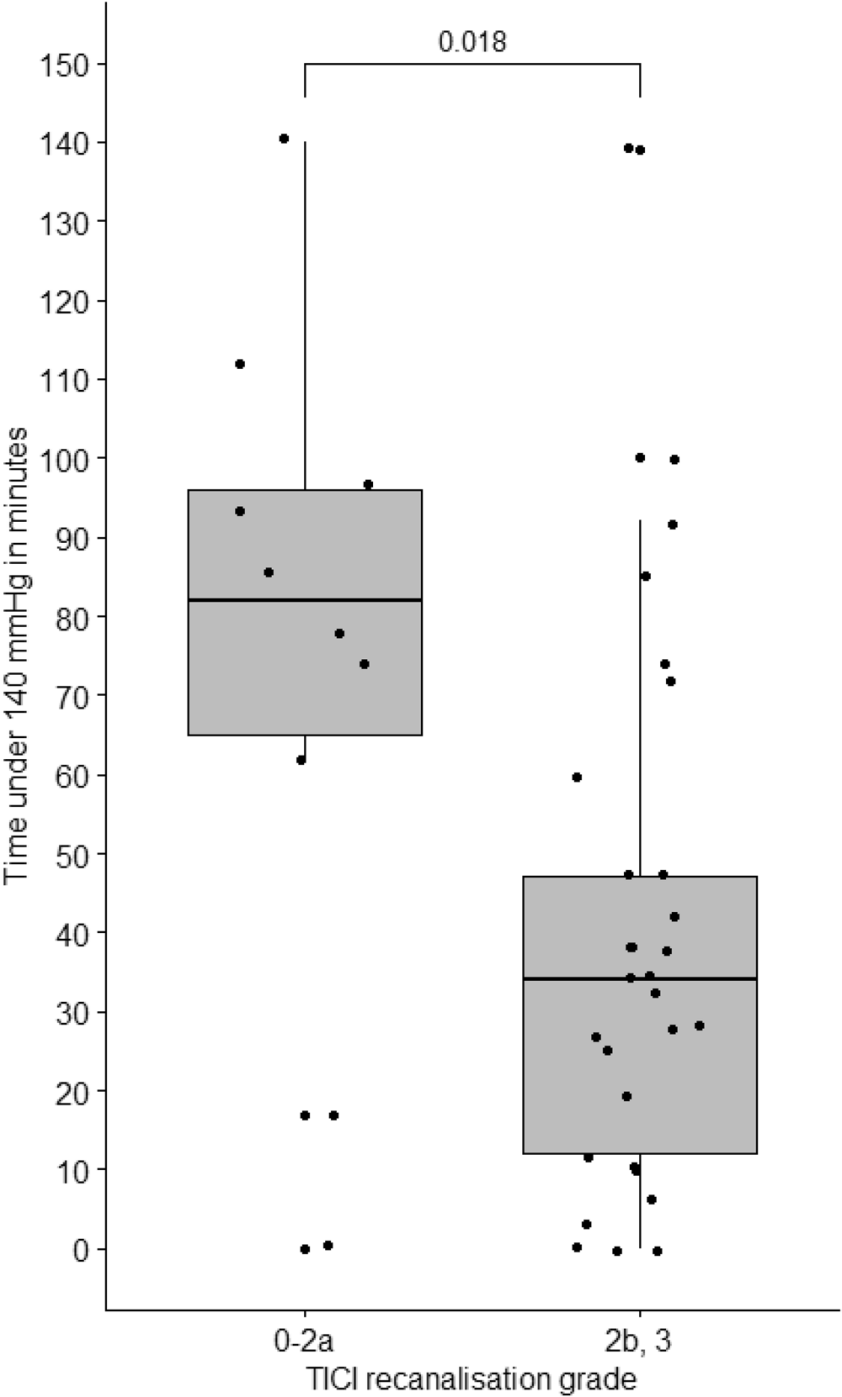
Success of recanalization in 39 patients treated with endovascular thrombectomy for acute basilar artery occlusion as assessed by Thrombolysis in Cerebral Infarction (TICI) grading scale.

We found that patients with failed or insufficient or failed recanalization (TICI 0–2a) were exposed to SBP lower than 140 mmHg for a significantly longer time than in patients with successful recanalization (TICI 2b-3). The median time below 140 mmHg was 31 (IQR 13.8–49.0) minutes for recanalized and 82.0 (65.0–96.0) minutes for non-recanalized occlusions (p = 0.009). Details are shown in Fig. 2.

**Figure 3:**
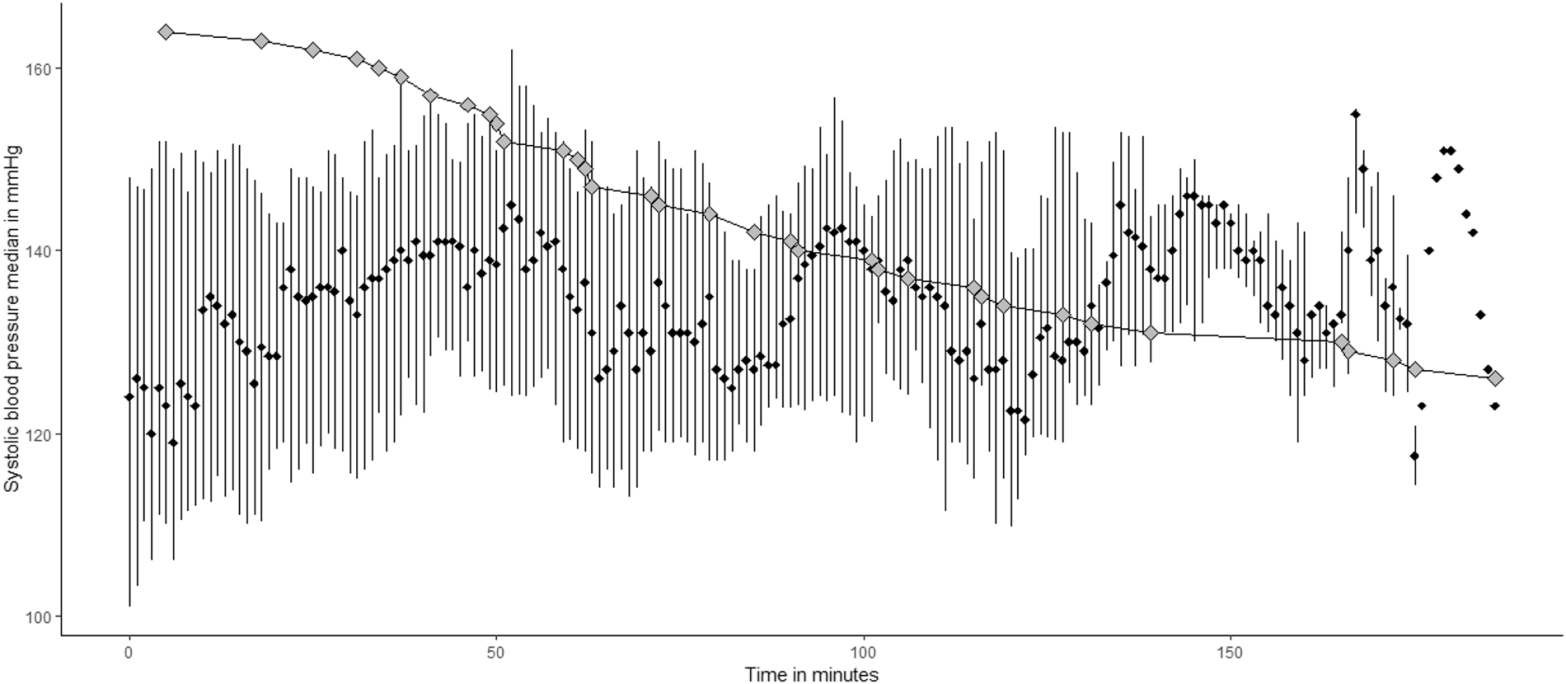
Systolic blood pressure during EVT for posterior circulation ischemic stroke. The black diamonds represent the median, the whiskers are the 25% and 75% percentiles. The grey diamonds account for the number of patients under intervention which is n = 39 at the beginning and n = 0 at 168 minutes. Of note, the first EVT was finished after 5 minutes and the last one at 168 minutes.

Figure 3 shows how median SBP evolves in the course of EVT. We could identify three distinct peaks, up to an SBP of 160 mmHg. The first SBP surge precedes the end of EVT in a subgroup of patients, then approximates to values at the start of the procedure. There are 2 more surges associated with extended procedure durations.

### Correlations between BP measures

We found significant positive correlations between EVT duration and SBP time under 140 mmHg and SBP time 20% above the reference value (p < 0.001, OR 0.55 95% CI 0.28–0.73).

There was a significant correlation for both number of SBP excursion and duration of time spent 20% below reference SBP with the number of excursions and duration of time spent over 180 mmHg (p < 0.01, OR 0.45, 95% CI 0.16–0.67), which reflects the presence of SBP variability.

### Outcome

Patient demographics and different clinical and paraclinical variables dichotomized for good and poor outcome are shown in Table 4. Outcome did not differ for BA or TBA occlusion. Furthermore, outcome was not distinct if iv-tPA was administered. The rate of recanalization outcomes as studied by the TICI score was not statistically distinct in patients with good vs. poor outcome. Good outcome was associated with conscious state on admission. There was a higher rate of intubated patients in the cohort with poor outcome (68.8%) than in the group with good outcome (8.7%, P < 0.001). In addition, there were more patients with pre-MRS of 0–2 (p = 0.033) and lower NIHSS (p < 0.001) in the group with good outcome. Presence of brainstem, cerebellar or thalamic infarction as well as hemorrhage on follow-up imaging increased the likelihood of poor outcome.

**Table 4:** Patient demographics and blood pressure dichotomized for good and bad outcome.

	Total N = 39	Poor outcome, N = 16	Good outcome, N = 23	P
Age (years)	73.5 (59.3–81.5)	76.2 (69.5–82.8)	72.8 (56.1–77.7)	0.098
Pre-mRS 0–2	33 (84.6)	11 (68.8)	22 (95.7)	0.033
Glucose on admission	130.0 (115.0–152.0)	141.0 (126.8–157.2)	123.0 (111.5–138.0)	0.098
NIHSS at admission	16.0 (6.5–42.0)	42.0 (28.0–42.0)	8.0 (5.0–15.0)	<0.001
BA occlusion	26 (66.7)	13 (81.2)	13 (56.5)	0.169
IV t-PA	22 (56.4)	8 (50.0)	14 (60.9)	0.531
General anesthesia	35 (89.7)	12 (75.0)	23 (100.0)	0.022
Intubated	13 (33.3)	11 (68.8)	2 (8.7)	<0.001
**SBP indices**				
SBP standard deviation	11.8 (7.3–16.5)	10.0 (8.2–15.1)	12.6 (7.3–16.8)	0.732
SBP coefficient of variation	8.7 (5.5–12.8)	6.9 (5.4–12.8)	9.2 (5.7–12.8)	0.689
SBP average real variability	3.6 (2.5–5.1)	3.7 (2.3–4.6)	3.3 (2.6–5.1)	0.819
SBP maximal-minimal difference	59.0 (28.5–78.5)	46.5 (33.2–74.2)	65.0 (28.0–78.5)	0.627
Number over 120% of reference SBP	0.0 (0.0–13.0)	4.0 (0.0–42.2)	0.0 (0.0–10.5)	0.225
Time over 120% of reference SBP	0.0 (0.0–15.0)	4.0 (0.0–42.2)	0.0 (0.0–10.5)	0.237
Time under 80% of reference SBP	0.0 (0.0–1.5)	0.0 (0.0–0.2)	0.0 (0.0–3.0)	0.453
Number under 80% of reference SBP	0.0 (0.0–1.5)	0.0 (0.0–0.2)	0.0 (0.0–3.0)	0.453
Number over 180 mmHg	0.0 (0.0–2.0)	0.0 (0.0–2.0)	0.0 (0.0–1.5)	0.918
Time over 180 mmHg	0.0 (0.0–2.0)	0.0 (0.0–1.2)	0.0 (0.0–2.0)	0.785
Number under 140 mmHg	37.0 (14.5–75.0)	53.5 (37.0–87.8)	27.0 (11.5–61.0)	0.116
Time under 140 mmHg	38.0 (18.0–76.0)	54.5 (38.0–87.8)	28.0 (14.5–67.0)	0.092
Number under 100 mmHg	0.0 (0.0–3.5)	0.5 (0.0–5.2)	0.0 (0.0–3.0)	0.608
Time under 100 mmHg	1.0 (0.0–5.0)	1.5 (0.0–6.0)	0.0 (0.0–4.5)	0.386
**Drugs administered**				
BP augmentation drug given	20 (51.3)	5 (31.2)	15 (65.2)	0.054
BP lowering drug given	2 (5.1)	1 (6.2)	1 (4.3)	1.000
**Neuroradiological outcome**				
TICI 0–2a	10 (25.6)	6 (37.5)	4 (17.4)	0.264
Presence of bleeding	6 (15.4)	6 (37.5)	0 (0.0)	0.015

BP – blood pressure, pre-mRS – modified Rankin scale prior to stroke onset, NIHSS – National Institutes of Health stroke scale, BA – basilar artery, SBP – systolic blood pressure, TICI – thrombolysis in cerebral infarction scale. Data are number (percentage) or median (interquartile range).

The rate of intraparenchymal hemorrhage on follow-up imaging was higher in patients with excursions and duration of time with SBP > 180 mmHg (both p = 0.029) and with SBP above 20% of the individual reference RR (p = 0.038).

Time spent with RR < 140 mmHg prior to intervention was negatively correlated with good clinical outcome. We found that patients with good outcome spent a median of 28.0 minutes with a SBP < 140 mm Hg (IQR 13.2–65.0), whereas the duration was 62.0 minutes (IQR 40.0–89.5) in patients with poor outcome (p = 0.03).

There was no correlation for intra-procedural SBP and MAP values with clinical outcome. However, pre-interventional mean SBP was significantly higher in patients with good outcome (mean 134.3 vs. 123.8 mmHg p = 0.03).

## Discussion

In this study, we describe in patients with acute BAO a steady increase of BP during EVT by using high resolution monitoring and identified temporal patterns of BP peaks and troughs. Despite a moderate increase of BP during intervention, more than half of the patients required BP augmentation by using sympathomimetic drugs to maintain blood pressure within the target range for the MAP of 65–130 mmHg. In addition, we found that patients with isolated TBA occlusion had significantly more BP excursions beyond 20% of the reference SBP and required more frequent use of sympathomimetic drugs compared to vertebrobasilar artery occlusion. Interestingly, successful recanalization was more common in patients with a shorter intra-procedural duration of SBP under 140 mmHg. We also demonstrate that there is a higher chance of good outcome with higher SBP measured prior to EVT. In contrast, brain hemorrhage was more frequent in patients with intra-procedural SBP episodes 20% above of the individual reference value and a longer duration with SBP above 180 mmHg.

There are only a few studies using invasive and/or high-resolution measurements, which reported temporal evolution of BP during acute ischemic stroke and more specifically EVT. A previous study measured BP non-invasively with a median number of 26 recordings (range 18–33)^17^, which contrasts our study with predominantly invasive arterial data from a median of 72 (QR 39.5–116.5) measurements. We therefore cannot compare our findings directly with the aforementioned study, which also did not exclusively deal with stroke in the posterior circulation. Yet, it is interesting to see a similar range for SBP and MAP (135 vs. 146 and 92 vs. 80 mmHg for SBP and MAP, respectively). We would like to emphasize that while there were more patients with successful recanalization and intra-interventional time spent under 140 mmHg, we can only speculate about the potential reasons for this finding. Notably, it was believed until recently that BP increases in acute ischemic stroke as a consequence of disturbed autoregulation, damage or compression of brain regions that control the autonomic nervous system and neuroendocrine factors^25^. Fischer *et al*. could refute this dogma by comparing premorbid BP in major ischemic stroke in a large population-based study^26^. They found that acute-phase SBP in these patients was much closer to the accustomed long-term premorbid level. With regard to EVT, decreases from baseline BP and hypotension during the intervention have been therefore found to be detrimental^27^. Brain territories responsible for BP variability have been investigated in several studies, and recently Kitamura *et al*. showed that BP variability in the hyperacute phase tended to be low in patients with right insular infarction and high in those with left insular infarction^28^. However, this effect persisted only in the hyperacute phase for the first 24 hours after admission.

There was a steady increase of BP during EVT compared to pre-procedural values but need to acknowledge confounders. Indeed, this finding could be explained by inverse effects by the use of vasopressors on the one hand, and impact of GA, which is known to lower BP, on the other hand. We found a similar pattern of sympathomimetic medication usage in our previous study evaluating BP during EVT in anterior circulation ischemic stroke^15^. Since both cohorts were treated almost exclusively under GA, the pattern of vasopressor support should deserve more attention in future studies. Differences with regard to BP regulation during recanalization therapy for vertebrobasilar and TBA occlusion may be related to damage or overactivation of critical brain regions. The rostral ventrolateral medulla (RVLM) is associated with cardiac and vasomotor regulation and compression of RVLM can cause activation of the sympathetic nervous system and the development of hypertension^29^. Moreover, in stroke involving the brainstem and particularly the pons, an increase of BP has been reported in contrast to injury to more rostral regions^30^. Indeed, compared with our previous study which evaluated arterial SBP and MAP values for EVT in acute anterior circulation large vessel occlusion (LVO), intra-procedural mean SBP in BA occlusion was higher (135 vs. 128 mmHg), whereas MAP was comparable (92 vs. 91 mmHg)^15^. Both cohorts adhered to in-house intraprocedural BP management protocols which aimed at avoiding severe hypotension.

We could show that good outcome was more likely in patients with higher SBP and MAP before the start of EVT. This could be due to, as suggested by others, of maintaining penumbra viability with sufficient albeit individual BP. However, in this study intra-procedural mean SBP and MAP were not associated with outcome. Notably, Schönenberger *et al*. found an association of reduced 24-hour NIHSS with lower diastolic BP. However, they could not find, at least in anterior circulation stroke, an association of intra-procedural BP indices and clinical outcome^17^. A recent study reported that a lower BP within the first 24 hours after EVT had positive impact on outcome, which further illustrates the complex interaction of BP at different time points and outcome in severe ischemic stroke^31^. Interestingly, in that study the rate of symptomatic ICH did not differ between the patients with a median of SBP < 140 mmHg and ≥140 mmHg, which could point at a higher relevance of intra-procedural compared to post-interventional phase for the development of hemorrhagic complications. Indeed, intracerebral bleeding could be due to procedural complications such as vessel perforation or reperfusion injury. Moreover, we previously reported a role of the immune system for the occurrence of hemorrhage in patients treated with EVT for anterior circulation ischemic stroke^32^. We detected intracranial hemorrhage in four patients (10.3%) and corroborate previous studies which reported an association of intracranial hemorrhage and bad outcome^2^. This rate is higher than reported in the study by Kang *et al*. (1.9% symptomatic), which also studied BA occlusions but used stricter inclusion criteria^28,33^. In addition, we confirm that BP surges are a risk factor for the development of hemorrhage in EVT for BAO stroke. We postulate that stricter attention to SBP over 180 mmHg may reduce the rates of symptomatic hemorrhage.

Management of BP in acute ischemic stroke is a tightrope journey where benefits and harms still need to be defined. We reported recently a U-shaped relation of BP with outcome in anterior circulation ischemic stroke^34^. Thus, patients with lower and higher BP values have a higher frequency of unfavorable outcome. Another study found that recanalization interacts with BP within the first 24 hours of acute ischemic stroke, and subsequently influences outcome^14^. The patients experiencing recanalization had a linear relationship with BP, with lower BP being associated with better outcome. In contrast, non-recanalized patients had a J-relationship, meaning those with lowest and higher BP have a less favorable prognosis. We found a similar relationship in our cohort, as patients with surges above 180 mmHg developed bleeding and patients with relative pre-procedural hypotension had worse outcome. Our cohort was too small to assess for the interaction of BP and recanalization. In addition, there is emerging evidence for the impact of BP post intervention.

We acknowledge that study has all the limitations of a single center retrospective analysis and limited number of patients. We also did not consider non-invasive on admission BP in our analysis and post-procedural imaging was not performed in a standardized time frame and was mostly done with CT. Eventually, we did not have a control group, and therefore we cannot compare most of our multimodal BP parameters.

## Conclusion

In conclusion, our study demonstrates complex dynamics of pre- and intra-procedural BP and the frequent need for medical augmentation of BP during intervention. While we found that intracranial hemorrhage is associated with an intra-procedural surge of BP, higher pre-procedural SBP was correlated with better clinical outcome at discharge. These findings point to a dysregulation of the vegetative nervous system in ischemic stroke in posterior circulation ischemic stroke even more than in large vessel occlusion of the anterior circulation. Additional well-designed studies are required to independently validate our initial observations, determine patients at risk for BP variability and subsequent poor outcome and impact of more aggressive BP management.

### Compliance with ethical standards

The study protocol was in accordance with the guidance of our hospital’s committee for the protection of human subjects (protocol UN 2553) and approved by the local ethics committee (415-EALL/5/32-2019). According to national regulations, a written approval of individual patients is not required for the anonymized analysis of routinely collected clinical and radiological data as used in this study.

## Data Availability

Individual patient data can be provided upon reasonable request.
